# Cooperativity within proximal phosphorylation sites is revealed from large-scale proteomics data

**DOI:** 10.1186/1745-6150-5-6

**Published:** 2010-01-26

**Authors:** Regev Schweiger, Michal Linial

**Affiliations:** 1 School of Computer Science and Engineering, Hebrew University of Jerusalem, 91904, Israel; 2 Department of Biological Chemistry, Institute of Life Sciences, Sudarsky Center for Computational Biology, Hebrew University of Jerusalem, 91904, Israel

## Abstract

**Background:**

Phosphorylation is the most prevalent post-translational modification on eukaryotic proteins. Multisite phosphorylation enables a specific combination of phosphosites to determine the speed, specificity and duration of biological response. Until recent years, the lack of high quality data limited the possibility for analyzing the properties of phosphorylation at the proteome scale and in the context of a wide range of conditions. Thanks to advances of mass spectrometry technologies, thousands of phosphosites from in-vivo experiments were identified and archived in the public domain. Such resource is appropriate to derive an unbiased view on the phosphosites properties in eukaryotes and on their functional relevance.

**Results:**

We present statistically rigorous tests on the spatial and functional properties of a collection of ~70,000 reported phosphosites. We show that the distribution of phosphosites positioning along the protein tends to occur as dense clusters of Serine/Threonines (pS/pT) and between Serine/Threonines and Tyrosines, but generally not as much between Tyrosines (pY) only. This phenomenon is more ubiquitous than anticipated and is pertinent for most eukaryotic proteins: for proteins with ≥ 2 phosphosites, 54% of all pS/pT sites are within 4 amino acids of another site. We found a strong tendency for clustered pS/pT to be activated by the same kinase. Large-scale analyses of phosphopeptides are thus consistent with a cooperative function within the cluster.

**Conclusions:**

We present evidence supporting the notion that clusters of pS/pT but generally not pY should be considered as the elementary building blocks in phosphorylation regulation. Indeed, closely positioned sites tend to be activated by the same kinase, a signal that overrides the tendency of a protein to be activated by a single or only few kinases. Within these clusters, coordination and positional dependency is evident. We postulate that cellular regulation takes advantage of such design. Specifically, phosphosite clusters may increase the robustness of the effectiveness of phosphorylation-dependent response.

**Reviewers:**

Reviewed by Joel Bader, Frank Eisenhaber, Emmanuel Levy (nominated by Sarah Teichmann). For the full reviews, please go to the Reviewers' comments section.

## Background

A large fraction of eukaryotic proteins undergo post translational modifications (PTMs) [1]. These PTMs, that are often restricted in time and space, occur in response to changing cellular conditions. Most eukaryotic proteins are subjected to several PTM types [2], however, the transient nature of PTMs poses a technological challenge in respect to their identification and quantification [1,3,4]. The most studied PTM is probably phosphorylation by protein kinases. In humans, there are over 500 kinases and ~150 phosphatases [5]. The phosphorylation status of a protein reflects a balanced action between protein kinases and phosphatases [6]. It is estimated that ~30% of cellular proteins from yeast to humans are candidates for phosphorylation on Tyrosine (Y) Serine (S) and Threonine (T) residues.

From a cellular function perspective, phosphorylation may lead to a transient change in catalytic activity, structural properties, protein turnover, lipid association, clustering, protein-protein interaction, translocation and more [7]. It is believed that a combination of phosphorylation events are often translated into cell decisions, as in the cell cycle [8], apoptosis [9], inhibition of translation [10], transcription [11] and even learning and memory in neurons [12].

Previous works have shown that multi-phosphosites are not randomly spread along the protein length [13,14] but instead are concentrated in protein surface patches [15,16]. Recently, the properties of phosphorylation clusters were analyzed in the context of additional types of PTMs [17]. It was shown that the co-occurrence of multiple phosphosites enable the execution of desired outcomes (e.g., complex assembly, protein-protein interaction, substrate dephosphorylation, subcellular localization and integration of pathways) [2]. While it is common for many eukaryotic proteins to have multiple phosphosites, the order by which these sites become activated or the duration of time that such sites remain phosphorylated are enigmatic (discussed in [18-21]).

Until recent years, the lack of high quality data limited the possibility for analysis on a phosphoproteome scale [19]. The growing body of mass spectrometry (MS) data and the improvement of phosphorylation detection methodologies [18,22,23] provide an opportunity to search for emerging properties in phosphorylation sites (phosphosites) and to challenge their functional relevance. We set out to perform a statistical assessment of phosphosites distribution along the polypeptide chain of eukaryotic proteins. We find that many phosphosites are characterized by a unique positional distribution. We show that clusters of phosphosites are evident for pS and pT but not pY sites. In addition, we show that closely positioned sites tend to be activated by the same kinase. Finally, we show that activating phosphosites within a cluster tends to be coordinated and strongly dependent. The implication of our findings on cellular regulation and on the advantage of such a property is discussed.

## Results

**Figure 1:**
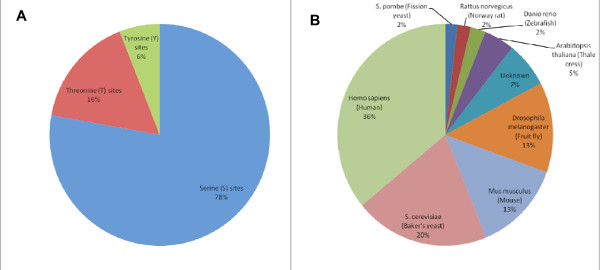
**Statistics of phosphosites origin and types**. **(A) **Analysis of the different types of phosphosites complied from SysPTM, Phospho.ELM and PHOSIDA. (**B**) The distribution of phosphosites according to their organisms. Organisms that have less than 1% of the total phosphosites are not shown. It accounts together for less than 1%. See Table 1 for further information.

MS proteomics data was subjected to statistical analysis with the goal of extracting hidden trends at a phosphoproteome scale. Currently, about 70,000 phosphosites have been reported. The unavoidable duplication in different databases was resolved by collapsing identical sequences into a single entry (see Methods). Figure 1 shows the phosphoproteins that were included in the analysis. The phosphoproteins represent an inclusive collapsed list from 10 different high quality resources. Major datasets include UniProtKB, Phopsho.ELM and PHOSIDA. The majority of the proteins from this set are mammalian (mostly human and mouse) though ~20% of the proteins are from yeast and a similar fraction is from the fly phosphoproteome.

Throughout all analyses, we separated Serine/Threonine (S/T) phosphosites from Tyrosine (Y) phosphosites. The S/T residues were treated collectively in accordance with the mode of activation by the relevant kinases [24,25]. Analyses that was carried out separately for pS and pT show that their properties are generally not significantly different, confirming the validity of such a partition (Figure 1, Table 1).

**Table 1:** Number of phosphoproteins and phosphosites included in this study.

Organism^a^	Number of Proteins^a^	Number of Sites	Average Site/Protein
Rattus norvegicus (Rat).	187	89	0.48
Schizosaccharomyces pombe (Fission yeast).	925	499	0.54
Rattus norvegicus (Norway rat).	1029	470	0.46
Danio rerio (Zebrafish).	1137	686	0.60
Arabidopsis thaliana (Thale cress).	2315	1294	0.56
Unknown	3410	1639	-
Drosophila melanogaster (Fruit fly).	6709	1793	0.27
Mus musculus (Mouse).	6773	2938	0.43
Saccharomyces cerevisiae (Baker's yeast).	10297	2459	0.24
Homo sapiens (Human).	18311	6023	0.33

^a^Only organisms with >100 known phosphoproteins are listed.

### S/T Phosphosites are Clustered, Y Phosphosites to a much Lesser Extent

It has been observed in many studies that phosphosites tend to appear in clusters [16,17,26,27]. The phenomenon of clusters of phosphorylation was exhaustively studied for several protein families such as the cyclin-dependent kinase (CDKs) [13,14]. Despite the numerous detailed reports on phosphorylation clusters, the universal nature and scope of these observations was not examined on the scale of the entire phosphoproteome.

**Figure 2:**
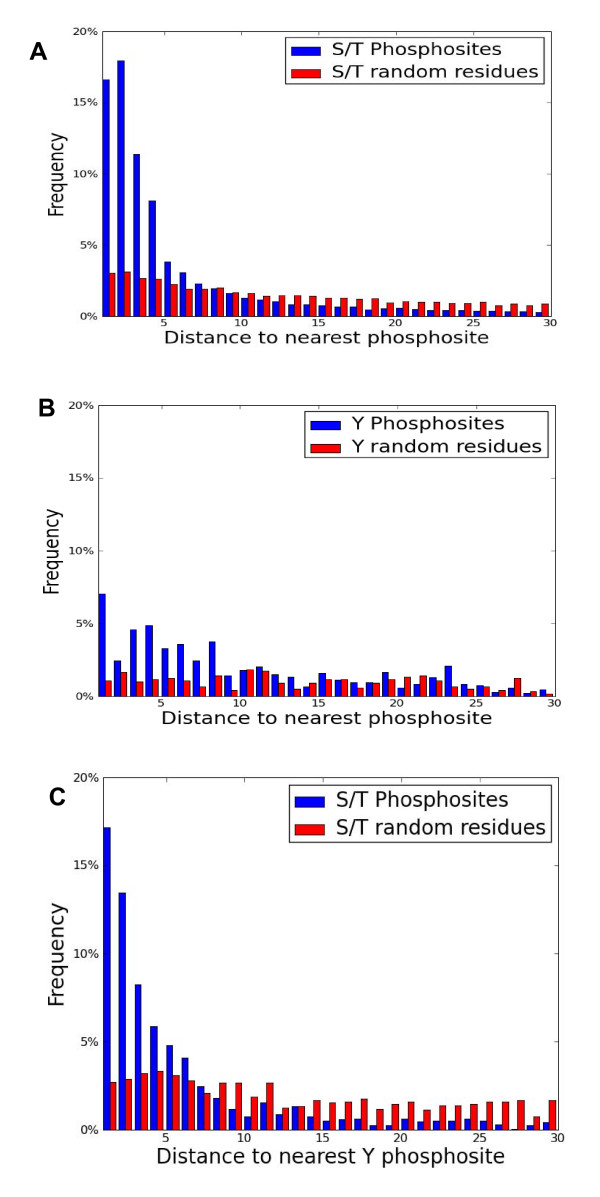
**Distances of nearest phosphosites**. **(A) **Analysis of ~51,000 non- redundant S/T phosphosites from unique proteins (**B**) Analysis of ~3160 non-redundant Y phosphosites. For each distance, the frequency is shown relative to the frequency of randomly selected from the relevant amino acids (see Methods). (**C**) Analysis of S/T phosphosites as in A, the distance to the nearest Y phosphosite is reported. The tail distribution of phosphosites including a distance >30 amino acids is provided in Additional file 5.

We examined the distribution of distances between adjacent phosphosites for the set of all known phosphoproteins (in units of amino acids; e.g., two sites with a distance of 1 are adjacent). For each phosphosite we take the distance between itself and its closest neighbor (namely, the minimum of the distances between itself and its 2 closest neighbors in the protein sequence, if they indeed exist). Figure 2 shows such a histogram. 45% (~10,700) of all phosphoproteins have only a single phosphosite and are excluded from this analysis. As a control, we created a background distribution that consists of random residues and measurement of their mutual distances (see Methods, Figure 2).

Figures 2A, B show that the local distances for all S/T sites (51,124 phosphosites) are distributed differently than Y phosphosites (3160 phosphosites). Statistically, using a 2-sample Chi square test, the difference is found to be significant (p-value < 1.0e-299). This difference cannot be attributed to the relatively small number of Y sites (~6% of all sites). For pS/pT and pY histograms, the differences from the background distributions (Figure 2, marked in red) and the occurrence of the relevant phosphosites are also very significant (p-values < 1.0e-299 and 3.6e-42 respectively).

It was shown that phosphosites tend to belong to disordered regions (see [28]). It would have been possible to conclude that phosphosites clustering is a mere result of the fact that phosphosite generally reside in limited regions. As a more stringent examination, we performed the comparison to a background distribution that takes into consideration the proportion of sites inside and outside disordered regions (see Materials and Methods). Although the background distribution is indeed somewhat different, the difference in the results is negligible.

To test whether the clusters of pS/pT and those of pY are excluded, we examine the distance between an S/T phosphosite and its nearest Y phosphosite (if such exists). Figure 2C shows that indeed Y phosphosites tend to be clustered to S/T phosphosites (~2000 sites, p-value < 1.0e-320). The average distance between two adjacent pS/pT sites is ~46 amino acids, while the average distance between a pS/pT site and its closest Y phosphosite is ~66 amino acids; thus, clustering between S/T sites is stronger than with Y sites. We conclude that the S/T phosphosites display a strong tendency to cluster with other phosphosites that is not reflected by the mere distribution of the amino acids (S, T and Y), and that this appears to be a general phenomenon.

Figure 2A shows that over 54% of all S/T phosphosites analyzed have an adjacent S/T site detected within 1-4 amino acids. The most prevalent distance is 2 amino acids. A similar analysis for Y-phosphosites shows that only 19% of the sites are found within this 1-4 amino acids range from another Y site. Both distributions display a long tail, where only 20% of S/T sites have a distance greater than 30 (10% above 100, 0.4% above 1000) while 45% of Y sites have a distance greater than 30 (25% above 100, 10% above 300, 0.4% above 2000).

**Figure 3:**
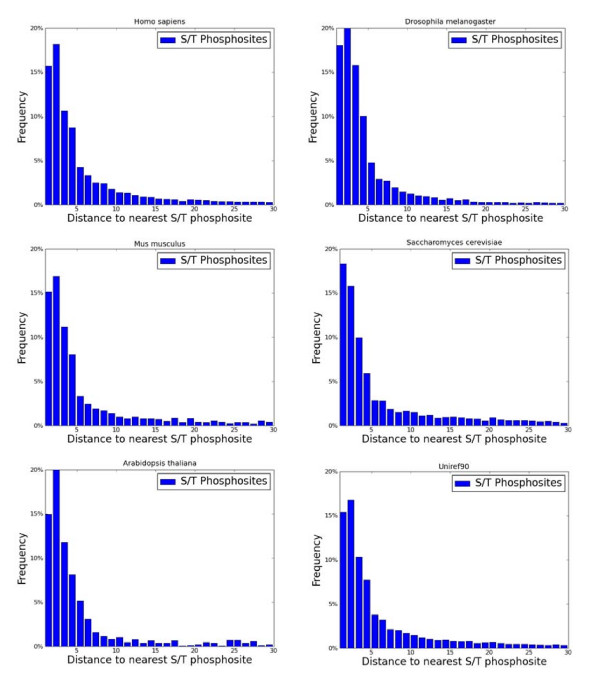
**Distances of nearest phosphosites partitioned by model organisms and non redundant sequences**. Analysis of ~51,000 phosphosites was performed as in Figure 2. The data were separated according to major organisms including human, mouse, Drosophila, Arabidodpsis and yeast. In all organisms, 32-37% of the pS/pT sites are within a distance smaller than 3. The data from UniRf90 show the reduction of UniProtKB phosphoproteins to a non-redundant set in which no two proteins share more than 90% sequence identity. Results from the non-redundant set (UniRef90) are identical to the complete set.

To ensure that the data is not heavily biased towards certain sets of proteins, we repeated the analysis for: (i) sets of proteins of different taxonomic origins (human, mouse, fly, plant and yeast); and (ii) for datasets where sequence similarity has been filtered out at two thresholds (90% and 50%, from UniRef90/50, respectively). The results of these controls are shown in Figure 3.

We somewhat arbitrarily define "proximal phosphosites" as sites situated within 4 residues of other matching phosphosites (where pS/pT matches pS/pT and pY matches pY). We have used this definition for the rest of the analysis. Note that comparable results for the phenomena reported in this manuscript for "proximal phosphosites" were obtained with other choices for a threshold on the distance of neighboring sites (in the range of 1 to 5 residues, not shown).

In order to refine the observation of *proximal phosphosites *for S/T phosphosites, we tested if this trend is limited to two adjacent sites or whether this is a continuous effect. To this end, we created the statistics of pairs of distances between 3 consecutive phosphosites. If the distances were independent then we would expect, for each pair of distances X and Y, to appear as the multiplication of the frequencies in which we have seen X and Y in the set of distances. This defines a statistical model which we can compare our results to. Note that too many or too little appearances of pairs of distances are informative (see Methods for an explicit definition, Table 2).

**Table 2:** An analysis of patterns of 2 distances (in amino acids) between 3 adjacent S/T phosphosites.

Pair of Distances		Observed Count	Expected Count	P-Value	P-Value (Bonf. Correction)
More than expected					

1	1	493	310.7	1.1e-16	2.22e-14
2	2	530	436.7	6.9e-6	0.0013
2	1	429	368.4	0.00101	0.21
Less than expected					
3	2	203	295.5	6.1e-9	1.21e-6
4	1	123	185.9	5.3e-7	1.05e-5
4	2	166	220.4	7.3e-5	0.0145

Table 2 contains the most statistically significant pairs of distance where only results with p-value smaller than 0.01 have been reported. Distances have been checked up to a distance of 10 amino acids. It can be seen that the tendency to cluster is not a phenomena restricted to pairs of sites but instead, continues further for S/T phosphosites. Y phosphosites on the other hand did not show any statistical significance in this test.

### Proteins Rich in S/T Clusters are Functionally Distinct

**Figure 4:**
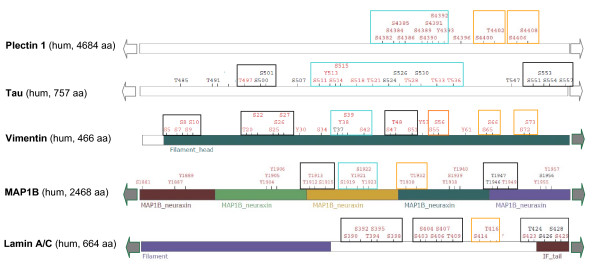
**A representative set of pS/pT clustered-rich proteins**. Short segments (75 amino acids each) that are exceptionally rich in clustered phosphosites are shown. These proteins have >5 *proximal phosphosites *clusters and >5 independent evidence from the literature. We marked clusters by a stringent definition where the distance between two consecutive pS/pT sites is at most n+3 (n denotes the position of pS/pT). The frames around the phosphosites denote the following: black, only one pair of pS/pT; orange, extended cluster according to the maximal distance of n+3 between neighboring pS/pT sites; blue, a mixed cluster of pS/T and pY. Phosphosites that are inferred from the identification of phosphosites in a close homologue are marked in a black font. For a complete list of clustered-rich proteins see Additional file 1

The statistical analysis shows that while 35% of phosphoproteins have at least one proximal phosphosite cluster, only 5% of the proteins have more than 5 such clusters. We set to study the exceptionally cluster-rich proteins in view of their functional assignments. As some phosphosites are weakly supported and may have resulted from faulty identification, we limited the analysis to proteins that have >5 independent supporting observations from the literature (Additional file 1). Figure 4 illustrates a focused view of 5 representatives from the exceptional cluster-rich proteins. Several observations are valid for these cluster-rich proteins: (i) most clusters are extended beyond the pair of phosphosites; (ii) pY sites are not excluded from the pS/pT clusters; (iii) the functions associated with the exceptionally cluster-rich proteins are dominated by structural proteins (cytoskeleton and intermediate filaments), signal transduction (membrane kinases, phosphatases and adaptors) and transcription regulators (transcription factors and mRNA processing) (Figure 4, Additional file 1).

### pS/pT Clusters Tend to be Phosphorylated by the same Kinase

We set out to test the behavior of kinase activity informed by our notion of proximal phosphosite clustering. We therefore asked whether *proximal phosphosites *tend to be phosphorylated by the same kinase. We used the compiled information from Phospho.ELM that specifies a list of kinases associated with many phosphosites. While a large fraction of the data originated from high throughput (HTP) experiments, 30% of the data are based on targeted experiments in which the identity of the reported protein kinase is confirmed.

We checked for each adjacent pair of phosphosites (for which the kinases are known) whether they could potentially be phosphorylated by the same kinase (defined as having at least one common kinase in the list of putative kinases). For the vast majority of phosphosites, there is only 1 such possible kinase (for a histogram of possible kinases for each site, see Additional file 2). Note that it is generally expected that a kinase will be reported as operating on multiple sites on the same proteins, especially as it is likely that a specific experiment might focus on one specific protein kinase, or a small family of protein kinases, which may introduce a bias towards concluding that being phosphorylated by the same kinase is preferable. We thus circumvented this potential bias by separating the analysis into two distinct sets - *proximal phosphosites *(as defined above), and all other sites (Table 3). We therefore examined whether being inside a phosphosite cluster affects the probability of being activated by the same kinase (Table 3, additional file 2).

**Table 3:** Activation of phosphosites by kinases.

S/T	Near phosphosites (distance < = 4)	Other phosphosites (distance > 4)
Same Kinase	393 (86%)	607 (62%)

Different Kinases	60 (14%)	365 (38%)

In general, it can be seen that adjacent sites tend to be activated by the same kinase. More importantly, division to *proximal phosphosites *emphasizes this tendency significantly (p-value of 1.25e-19). Repeating this analysis with Y phosphosites shows no statistical significance with respect to *proximal phosphosites*.

### S/T Phosphosites within a Cluster are Strongly Coordinated

An important aspect of phosphorylation regulation concerns the coordination between adjacent sites. Namely, whether the presence of a phosphate in a defined position accelerates or represses the presence of additional phosphates in adjacent sites. Phosphopeptides are the best source for such analysis. However, the variability in separation and elution protocols and evidently, the MS operational mode drastically affect the recovery, sensitivity and precision in identifying the position of the phosphosites [29,30]. We thus used several of the largest sets available that cover a wide range of technologies and a range of biological sources and experimental conditions. The results are based on a collective dataset of ~43,200 peptides from: (i) HeLa cells follow EGF stimulation, (ii) cell cycle, (iii) mouse liver cell line Hepa1-6, (iv) mitotic-arrested HeLa cells, (v) mouse liver and (vi) human non-small lung carcinoma cell line (H1299). As over 80% of all peptides consist of 6-16 amino acids, this analysis effectively focuses on *proximal phosphosites*. Many of the proteins are reported (with their respective sites) in multiple experiments.

**Figure 5:**
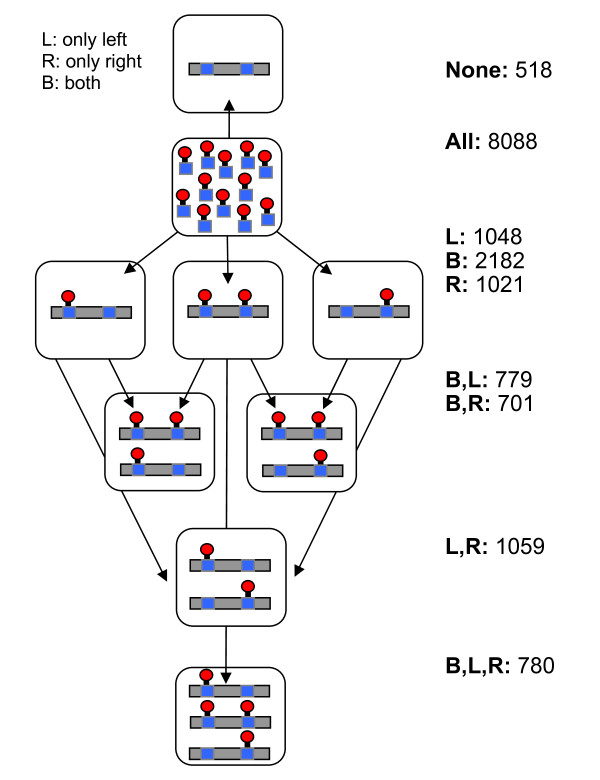
**Patterns in phosphorylation of adjacent phosphosites**. For each pair of phosphosites (from the entire sources for phosphoproteins), the peptides that contain both of them are searched. It is then asked if from these peptides, there are peptides that contain both sites in their phosphorylated state (marked as 'both', B), only the first site is phosphorylated (marked as 'left', L) or only the second site is phosphorylated (marked as 'right', R). Each pair of sites is assigned a pattern according to the types of peptides we have seen. For example, the rightmost bar contains pairs for which we have only seen peptides in which both sites are phosphorylated (marked only with B). Note that the amount of pairs not seen in any constellation is only ~5%, indicating a high coverage of the set of experimental results that were applied for this analysis.

Each peptide is reported with the exact phosphosites detected by MS. For each pair of consecutive potential sites, as reported by SysPTM [17], all the peptides containing the two sites were examined. These peptides were then divided into 3 distinct categories: (i) peptides where both sites were phosphorylated; (ii) peptides where only the first site of the pair was phosphorylated, and the second site was not; (iii) peptides where only the second site of the pair was phosphorylated, and the first one was not. For every pair of sites, we then ask if any peptides from each of the 3 categories were present in the data, assigning each pair an end result of one of 8 (2^3^) possible patterns (Figure 5).

The results show that the most dominant pattern is for the pair of sites that only appears together (Figure 5, marked B). This pattern represents a scenario in which the phosphorylation sites accumulate to reach a predetermined threshold.

The next prominent patterns are where from the pair of sites, only one appears phosphorylated in each peptide, where we have seen peptides with only the left site, with only the right site (Figure 5, marked L,R) and cases where we have seen either the left or right sides (Figure 5, L and R). These patterns are consistent with a scenario where a minimal set of phosphosites is needed for activation and their specific location is less critical. The trend in which both sites of a pair are phosphorylated (marked as B) was dominant also when individual experiments were analyzed separately.

### Features that Promote Protein Interactions are Augmented in Phosphosite Clusters

Based on the mtcPTM database [31] and on EGF-stimulation [32], it was shown that structural arguments are imperative in the accessibility of potential sites to their associated kinase. When accessibility was tested it was shown to be maximal for pS and somewhat weaker for pT [32]. A tendency for phosphosites to reside on exposed patches [16], coiled regions and disordered protein regions [28],Iakoucheva, 2004 #143] have been reported. Furthermore, phosphosites, display a tendency to reside outside globular domains [31,33].

**Figure 6:**
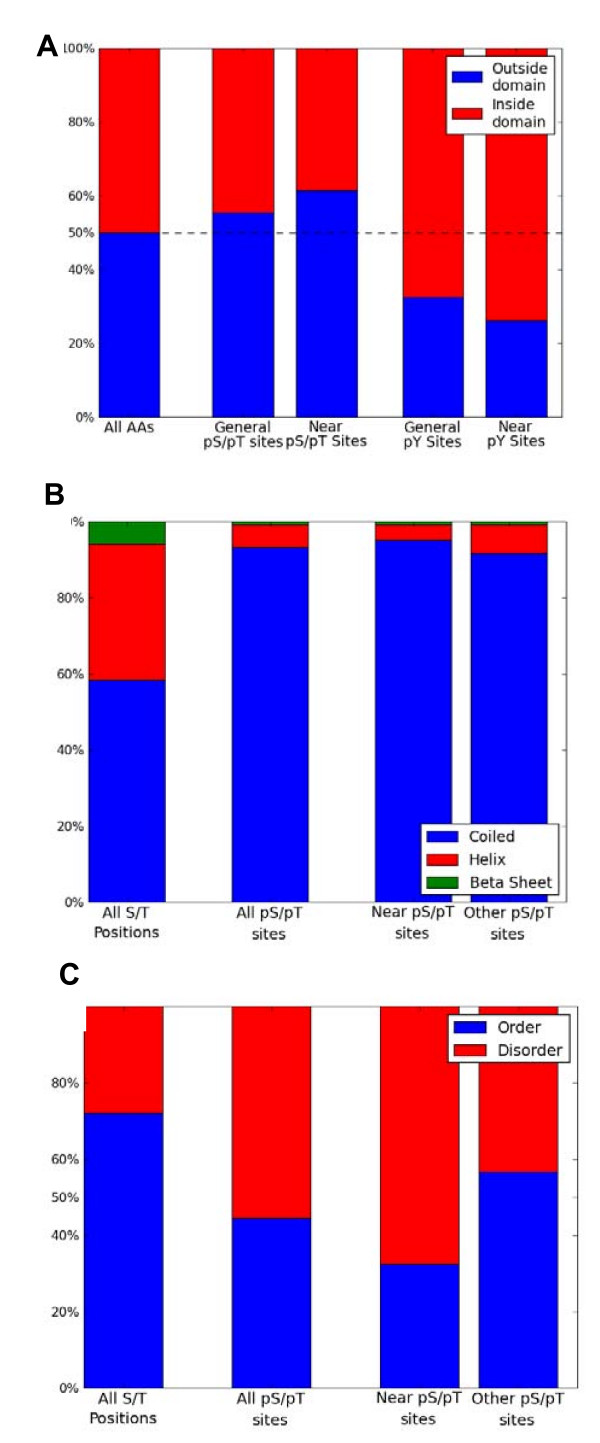
**Structural and biochemical features of pS/pT sites**. **(A) **The tendency of pS/pT sites to be inside/outside a domain. The proportions of being inside or outside a Pfam domain are measured for: (i) all amino acids, (ii) all S/T phosphosites, (iii) only S/T phosphosites with a near neighbor, (iv) all Y phosphosites and (v) only Y phosphosites with a near neighbor. (**B**) Distribution of secondary structure elements. The proportions of being coiled, in α-Helix or β-sheet for: (i) S/T positions that are not phosphosites (~12,000 random positions) (ii) all S/T phosphosites (~18,300 sites) where these are divided to: (iii) only S/T phosphosites with a near neighbor (~8400 sites) (iv) only S/T phosphosites without a near neighbor (~9900 sites). (**C**) Distribution of ordered and disordered elements. The proportions of being in disordered regions: (i) S/T positions that are not phosphosites (~36,700 random positions) (ii) all S/T phosphosites (~36,000 sites) where these are divided to: (iii) only S/T phosphosites with a near neighbor (~16,700 sites) (iv) only S/T phosphosites without a near neighbor (~19,200 sites).

We confirmed these properties, and observed that all of these tendencies increase when limiting the scope to the subset of proximal phosphosites. General S/T phosphosites tend to be outside of globular domains, with 55% of the phosphosites outside domains, and 45% inside. Examining only *proximal phosphosites *we obtained a more skewed set of values - only 38% of the S/T phosphosites reside within domains, with a p-value of 5.01e-5 (1105 sites, Figure 6A).

Similarly, in agreement to previous observations, phosphorylation sites tend to be in coiled regions (see Methods for secondary structure partition). A subtle difference is seen when the *proximal phosphosites *were separated from the rest of the S/T phosphosites (a significant difference of p-value 4.07e-21, Figure 6B).

Finally, it is evident that general S/T phosphosites display a strong tendency to be in disordered regions (p-value < 1e-299). However, further division according to clustering status shows that *proximal phosphosites *are significantly more likely to occur in disordered regions (68% relative to 43% for phosphosites that are at a distance ≤ 4 and >4, respectively, Figure 6C). The Y phosphosites still display a tendency to be in disordered region, although this is not as significant (p-value of 5.62e-15). More important to our discussion, the division to *proximal phosphosites *does not yield further insight for Y sites, displaying only a subtle difference from the distribution of all phosphosites (p-value of 0.002).

The increase in all previously observed structural and biochemical features (Figure 6) for proximal sites for pS/pT clusters but not for pY is consistent with a role of the pS/pT clusters in protein-protein interaction, while the pY sites are not necessarily optimal for this property(Figure 6).

## Discussion

In eukaryotes, the amino acids Serine (S), Threonine (T) and Tyrosine (Y) comprise ~15% of all protein sequences (7%, 5%, 3%, respectively). Yet, only sites that fulfill distinct biochemical or structural properties are subjected to phosphorylation by an arsenal of protein kinases. In recent years, large-scale studies, experimentally validated resources and literature curation became available for phosphorylation MS experiments [31,32,34]. Nevertheless, successful identification and reliable coverage of most phosphosites *in vivo *must still overcome technological and bioinformatics hurdles.

The systematic analysis we performed is based on the largest set of phosphosites available. Over 70,000 phosphosites were mapped to ~51,000 unique non repeated sequences. Within this set, large-scale *in vivo *and *in vitro *studies are combined. Note that numerous proteins share high similarity in sequence (i.e. homologues between human and mouse or paralogous genes). We choose to include closely related sequences (Figure 1), because phosphorylation sites tend to be little conserved, especially in disordered regions. Thus, even closely homologous proteins may still be informative and reveal global properties of their phosphosites (for quantitative arguments see [28,35]). Nevertheless, our results (Figure 2C) show that even when a representative set of the sequences are considered (i.e. UniProt90), the same quantitative properties of phosphosites clusters hold.

When phosphosites dependency is discussed (Figure 5), it becomes critical to separate individual experimental data and when available, rely on multiple, independent evidence. Still, high quality data remains the bottleneck for the phosphosites dependency observations. We expect that with advances in MS-based phosphoproteomics and the development of direct methods for large-scale phosphosites detection [23], the statistical power of our observation will increase.

### Evolution Robustness in pS/pT Clusters

The conservation of phosphosites throughout evolution had been thoroughly studied [28]. It was suggested that phosphosites are significantly more conserved relative to other S/T sites [27,32]. A systematic study of the human phosphoproteome relative to other model organisms suggested that the phosphosites are evolutionarily dynamic, although the evolutionary conservation of pS/pT versus S/T was not explicitly tested [35].Interestingly, constraints on pS/pT did not limit the polymorphism as measured by SNPs in human populations compared with non-phosphorylated residues [28,36]. Tyrosine phosphorylation conservation is consistent with positive selection where the reduction in pY is in association with an increase in cell type complexity [35].

We therefore propose that the multiplicity of sites within S/T clusters provides a basis for their evolutionary robustness. Specifically, if a function is linked to a cluster of sites rather than an individual site, then we expect dynamics of gain and lost of nearby phosphosites. Such model was recently proposed [37]. Through a comparative analysis of closely related species [35] and functional experiments, an estimate for the evolutionary forces that shape the pS/pT clusters is expected. We are currently testing the possibility that phosphosite within the proximal sites of a cluster, show a unique tendency of conservation (Schweiger and Linial, in preparation).

### Coordination in Executing Biological Functions: Two are Better than One

The observation that most pS/pT in proteins with multiple sites reside in clusters raised the question on the cellular implication of the phenomena. Despite a limitation in quantitative information and the many unknown parameters, theoretical and mathematical models for multiple phosphorylations were proposed [38-40]. For example, it was suggested that processivity in phosphorylation may alter the sensitivity and speed of a cellular response [41,42]. A mechanistic role for *proximal phosphosites *as a stepwise sensor and as a delaying timer was illustrated for Cdc4, a key component in the protein complex that determines cell cycle control [43]. Our results are consistent with a dependency between pS/pT sites that are in close proximity (i.e., Table 3, Figure 5).

Investigating the proteins with super-rich phosphosites clusters (Figure 4) provides hints on the role for *proximal phosphosites*. These proteins share a restricted number of biological functions (mostly cytoskeleton, structural proteins and those involve in RNA regulations, Additional file 1). A plausible idea for the role of *proximal sites *in DNA binding proteins concerns the electrostatic nature of the phosphosites. If the bulk electrostatic charge is the critical feature of the protein, the exact position of phosphosites is evidently less critical. Cytoskeleton proteins are abundant among the super-rich *proximal sites *cluster proteins. These proteins may benefit from having a gradual and additive threshold rather than an abrupt switching [41].

The results from Table 3 show that *proximal phosphosites *are mostly activated by the same kinase. The analysis is resistant to the apparent bias from experiments analyzing specifically only one or few protein kinases. Whether these events occur in parallel or in a sequential manner has yet to be determined.

While the results of Figure 5 lack a dynamic component, the support for coordination within a short region of adjacent phosphosites is evident. When phosphosites are considered 'quantitative', clustering of phosphates is beneficial. A mode where an ensemble of phosphosites provides a necessary platform was described [44]. Our analysis argues that the coordination property in phosphorylation is not attributed to pY but strongly supported for pS/pT sites.

Inspecting the Y phosphosites shows some tendency towards the prevalence of short distances. Actually, most of this signal originates from the instances associated with a specific Pfam domain family of the Tyr kinase catalytic domain (PF07714). An example is Jak3 kinase in which two adjacent tyrosines (Y980 and Y981) are located in the activation loop. Phosphorylation of each of these tyrosines affects Jak3 kinase catalytic activity. Repeating the analysis for S/T and Y phosphosites after eliminating the effect of Pfam kinase PF07714 resulted in diminishing the slight effect for pY with no effect on the S/T phosphorylation. The differences in distribution and biochemical features of pS/pT and pY agrees with the notion that pY-sites mostly serve as a discrete, on-off switch and thus their position may be more precise and possibly under tight control at the level of organisms and on an evolutionary scale [35].

Altogether, we show an analysis in which phosphosites clusters are appropriate statistical entities. Our results suggest that pS/pT clusters are the building blocks of phosphorylation regulation. When such clusters are considered, several of the known features that were noted in general phosphosites were augmented (i.e., pS/pT clusters in disordered regions and coils) while other are not validated (i.e., pY shows no evidence for cooperatively). Our global analysis provides a statistical view on the current collection of phosphorylation sites in view of the biochemical, functional and cell regulation properties in eukaryotic proteins.

## Conclusions

Until recent years, the lack of high quality data limited the possibility for analysis on a phosphoproteome scale. Based on advanced MS technologies, thousands of phosphosites from complex *in-vivo *settings were identified and archived in the public domain. Such a resource was used to statistically assess the phosphosites distribution in eukaryotes and their functional relevance. We show a strong prevalence of clusters of phosphosites throughout the evolutionary tree and thus it seems a far more general phenomenon than previously appreciated. Furthermore, we show that previously observed features of phosphosites are augmented in pS/pT clusters, but not in pY. We raise the notion of pS/pT clusters as the elementary building blocks in phosphorylation regulation. Under this assumption, we illustrate that closely positioned sites tend to be activated by the same kinase (86% of proximal pairs of phosphosites, compared to 62% of non-proximal pairs). Furthermore, a coordination and positional dependency is evident within proximal sites. We postulate that the unique design of pS/pT clusters is used to fulfill a range of cellular tasks.

## Methods

### Data collection

Data were collected and analyzed by considering phosphoproteins, phosphosites and MS phosphopeptides.

#### Phosphoproteins

Data regarding proteins, including their sequences, were acquired from UniProtKB (release 15.6) [45] and IPI (version 2.27) [46], NCBI Entrez Proteins [47], WORMPEP [48], TAIR [49], CYGD [50] and Flybase [51]. All sources were downloaded from the latest version available (as of July 2009). We used SysPTM to create a non-repeated protein set using rigorous identifiers mapping. SysPTM provides data for proteins from 10 different databases. We used the identifiers (IDs) mapping according to SysPTM (when available). We selected one protein out of each such overlapped group to avoid bias by duplication. When possible, we assigned the ID to the UniProtKB that provides the most reliable sequence information and annotations. Due to inconsistency in identifiers associated with each of the databases, and in order to reduce uncertainly, ~85% of the relevant proteins were successfully converted with a unified ID.

#### Phosphorylation Sites

We compiled an exhaustive set of phosphorylation sites based on SysPTM resource. SysPTM [17] was used as a source for a curated PTM database, from which we extracted only the phosphoproteins. The resource includes ~25,000 phosphoproteins with ~69,000 phosphosites. The data were collected from HTP experiments as well as from specific focused studies. We used the ID coverage from SysPTM, where such exist to match proteins obtained from different other resources. For matching protein kinases with phosphosites, we used Phospho.ELM (version 8.2) [34], which collects data from published literature as well as from HTP data sets. The positions of phosphosites for each protein and the corresponding protein kinases, where available, are extracted. Phospho.ELM includes ~4500 phosphoproteins with ~19,000 phosphosites. For high quality phosphosites identification we used PHOSIDA [32], which covers (i) Hela cell epidermal growth factor (EGF) stimulation [26]; (ii) kinase based study along the cell cycle [52] and (iii) mouse melanomas proteome analysis [53].

#### MS based Phosphopeptides

Data on phosphopeptides were analyzed from resources that are based on complementary technologies. Phosphopeptides from PHOSIDA were assigned identification scores as described [32]. Additional resources include: the mouse forebrain sample using affinity-based IMAC/C18 enrichment [54], the human mitotic phosphoproteome based on SCX chromatography, IMAC, and TiO_2 _enrichment [55], the mouse liver and Drosophila embryo [30]. All these datasets are assigned with identification confidence score [52,56]. We excluded studies that report on <1000 phosphopeptide identifications to avoid statistical biases that are due to experimental variability and high false positive rate. Only high confidence and non-ambiguous identifications were included for the analyses. We compared independent experiments that cover a major fraction of all reported phosphoproteins: (i) PHOSIDA HeLa cells that were metabolic tagged and following EGF stimulation at various time points with ~11,000 phosphorylation sites from ~2200 proteins [26] (ii) HeLa cells that were arrested in cell cycle with ~6200 unique sites of phosphorylation on ~1370 proteins [52] (iii) mouse liver cell line Hepa1-6 treated with phosphatases inhibitors, ~1800 proteins with ~5400 sites [57] (iv) mitotic-arrested HeLa cells following EGF activation, with ~13,300 phosphosites from ~3200 proteins [55] (v) mouse liver with ~5250 non redundant S/T phosphorylation sites from ~2150 proteins [58] (vi) human non-small lung carcinoma cell line (H1299), ~1300 proteins with ~2200 sites [59]. The data were available from the supplementary information of the publication and datasets for (i-iii) from PHOSIDA website [32]. False identification by MS on phosphosites and some ambiguous positioning is present in the raw data source. We excluded from the analyses all instances in which the exact position of the phosphosites is undetermined.

### Protein Annotations and Prediction Tools

Data regarding annotations are directly retrieved from UniProtKB [60]. Each protein is associated with a rich set of annotations that cover functional, structural, protein domain family assignment and sequence features. Data regarding the domain structure of proteins with UniProtKB ID [60] were acquired from the Pfam [61] site. The Pfam database (version 23.0) provides a collection of ~13,200 protein and domain families. For each protein, a mapping of all relevant domain families, the domain composition and domain architectures is provided. Each family is associated with rich functional and structural annotations include Gene Ontology [62], pathways and more.

#### Disordered Region Prediction

In order to identify areas of disorder, we applied DisEMBL [63]. We applied the predictor that was recommended by the authors with default parameters (Remark465).

#### Secondary Structure Prediction

For assigning secondary structure, we used PSIPRED [64]. PSIPRED classifies each residue into one of 3 classes: H (helix), E (extended β-sheet) and C (coil), assigning each one a level of confidence of 1-9.

### Statistical Analysis and Simulations

#### Random Selection of Positions for Background Distributions

Testing of various phosphosite properties for their tendency to be biased towards some classification (e.g., their tendency to be in globular/disorder regions) was performed. In addition, positional properties of the phosphosites were tested (e.g., their distance from near phosphosites). The analyses were performed by comparing the phosphorylated residues to the corresponding properties in random amino acid residues. When this was required, we randomly selected amino acid positions in the following way: (i) we calculated the empirical distribution of the number of phosphosites per protein (ii) from the non-redundant protein set, for each protein we selected at random an artificial number of random positions to choose, according to the distribution we have calculated (iii) we randomly selected several residues of the specific type (i.e. S/T or Y), in the number of random positions we have chosen.

A more stringent way to create such a random selection is to replace steps (i) and (ii) above with the process of simply taking the number of actual phosphosites on that protein, for each protein, as the number of random positions to choose., In addition, we also took the number of residues in ordered/disordered regions under consideration - for each protein, we first chose a number of residues from the disordered regions equal to the number of phosphosites on that protein that belong to the disordered region; then we similarly selected a number of residues from ordered regions. The results are essentially similar; the respective graphs for both methods are in the Additional Files (Additional files 3, 4).

#### Phosphosites Distances

Let us define *N*_*x *_as the number of times we have seen the distance *x *between two phosphosites, and N as the number of all distances we have seen also define *M*_*x*, *y *_as the number of times we have seen the pair of distances *x, y *between three adjacent phosphosites, and M as the total number of pairs of distances we have seen. If there was no dependency between two consecutive distances, we would expect *M*_*x*, *y *_to be binomially distributed - . We can therefore calculate a two-tailed test. The test results indicate (i) the probability of seeing the value of the specific *M*_*x*, *y *_or more, if we question whether there were significantly more such pairs or (ii) the probability of seeing the value of the specific *M*_*x*, *y *_or less, if we want to see if there were significantly less such pairs than expected. Each pair of distances provides then two p-values.

## List of Abbreviations

HTP: high throughput; MS: mass spectrometry; pT: phosphothreonine; pS: phosphoserine; pY: phosphotyrosine; PTM: post-translational modification; GO: Gene Ontology.

## Competing interests

The authors declare that they have no competing interests.

## Authors' contributions

RS performed the data collection and statistical analysis. ML wrote the initial draft of the manuscript and directed the study. RS and ML wrote together the final manuscript and designed the experiments. The authors read and approved the final version of the manuscript.

## Reviewers' Comments

### Reviewer's Report 1

Reviewer 1: Joel Bader, Department of Biomedical Engineering, John Hopkins Universit, USA

#### Reviewer's comment

This report analyzes the occurrence of phosphorylation sites (phosphosites) identified by mass spectrometry. The main conclusions are that pS/pT sites are clustered on proteins and clusters are often activated by the same kinase. In contrast, pY sites are not clustered. Fig. 1: The number of proteins (in addition to the fraction) should be displayed. It might be better to provide this information as a table, columns = types of phosphosites, rows = organisms.

#### Authors' Response

Such a table is now available as an added table (Table 1). We believe that showing the fractions for the organisms as in Fig. 1B is informative and support the claim on the generality of our observations. Therefore, we chose to keep the Fig. and add Table 1.

#### Reviewer's comment

On p. 6, "we take the minimum of the distances between itself and its 2 closest neighbors" - Is this the same as taking the distance to its closest neighbor? Distance should be specified as number of aa apart rather than 3D distance.

#### Authors' Response

It is indeed so; the manuscript was updated for clarification.

#### Reviewer's comment

On p. 6, A better randomization would be to randomize within each protein separately- a protein-by-protein control for analyzing the unequal/bunched distribution of S/T sites vs. Y sites. I think it would answer any complaints about confounding effects.

#### Authors' Response

Such randomization was performed as suggested. The two different random background distributions are essentially similar and therefore we have decided to keep our original formulation and include the suggested method in the additional files (Additional files 3, 4), with a respective note in the manuscript.

It should be noted that we in fact performed a more stringent randomization (as proposed by reviewer 3) that takes into account not only the number of sites in each protein, but also their positions regarding disordered regions, As can be seen, the two distribution are still very similar and therefore do not affect any of the conclusions. See detailed response to reviewer 3.

#### Reviewer's comment

Why is the figure truncated at distance 30? Why is there so much structure in the random residues results? Shouldn't there be a smooth decay similar to a negative binomial distribution?

#### Authors' Response

The reviewer is correct; there is nothing magical about distance 30. The truncation at distance 30 is arbitrary and is mainly done to put the focus on the more interesting part of the distribution.

As for the 'structure' in the random distribution: any evidence of structure is due to the number of samples for which we examine the resolution of the distribution. If we would have taken more samples, it would indeed disappear. Similarly, the random distribution indeed decays quite smoothly in a fashion similar to that of negative binomial/geometric distribution. An extension of both the real and random distributions for the pS/pT case was added to additional file 5 (for those taking interest in the distribution tail).

#### Reviewer's comment

It is probably important to correct for unequal occurrence of S/T and Y sites among proteins. Here is an idea: For each protein having S/T sites and Y sites, choose one S/T site and one Y site at random, and calculate the distance of these two selected sites to the closest other site. This generates a pair of values for each protein, and then a Wilcoxon paired signed rank test can be performed.

#### Authors' Response

While the chi-square test should not be affected by the size of the samples (unless too small, which is not the case here), we performed both this test and a test that randomly selects a subset of pS/pT sites in the size of the total number of pY sites, and calculates the 2-sample chi-square statistic. Both tests confirm these are indeed statistically different distributions.

#### Reviewer's comment

Table 1, P-values should be corrected for the number of distance pairs considered.

#### Authors' Response

Including corrections for multiple testing has a negligible effect on the significance of the P-values reported. We included an additional column for the Table (Table 2, revised) for the Bonferroni correction. It should be noted that even after this stringent correction, most of the P-values are still significant.

### Reviewer's Report 2

Reviewer 2: Frank Eisenhaber, Bioinformatics Institute A*STAR, Singapore

#### Reviewer's comment

In their initial part of the Results section, the authors provide statistical data that suggests clustering of pS/pT (but not pY) phosphosite clustering. At the same time, the question whether S/T sites in general have a trend to be more homogeneously distributed over the sequence remains unexplored (it is just stated in the first paragraph of the discussion).

#### Authors' Response

The distribution of general S/T sites over the sequence is indeed of interest and was previously studied by others. However, we chose not to focus on it in this study. The reason we could practically overlook this aspect is that we do not assume any homogeneousness of the distribution, since any comparison to general S/T residues is done using the empirical distribution. As this is a delicate issue, the discussion has been appropriately altered

#### Reviewer's comment

In a previous paper (Neuberger et al., Biology Direct, 2007, 2, 1), it was reported that PKA phosphosites tend to be surrounded by a region with a trend towards small, flexible and more polar amino acid residues. It appears likely that such regions are enriched in S/T residues and, thus, are more likely also to harbor multiple phosphosites. It can be that this enrichment is less pronounced that that of phosphosites.

#### Authors' Response

Thanks for the reference. Actually a comment with the same flavor was raised by reviewer 3 (see detailed response). The definition of flexible/polar region is to a large extent similar to the definition of 'disordered' regions. We thus refer to the 'disordered' regions as a more familiar definition for special regions in proteins.

#### Reviewer's comment

The amino acid compositional trends in the environment of phosphorylation sites also suggest a preference for more disordered regions of proteins. In the last part of the Results section, the authors explore the relationship of protein domains and phosphosites implying that the focus is to distinguish between sites in regions with well-defined 3D structure in comparison to more disordered parts of the sequence. It is known that many PFAM domains contain not only true globular domains but also transmembrane segments, signal peptides, flexible linker regions and the like. Thus, the trends observed by the authors should be much stronger if the domain library had been cleaned up for non-globular segments. The localization of a phosphosite in a flexible region is mechanistically important since the respective peptide segment needs to find a way into the catalytic cleft of the kinase.

#### Authors Response

We agree that the localization of phosphosites using a structural view is important and it was partially addressed by previous publications. Indeed, flexible regions are mechanistically of special importance. At present, Pfam does not provide an easy (or not easy) mechanism for partitioning domains to their globular/membranous etc. The application of such partition is feasible from additional resources. We consider this nice suggestion as a follow up study. However, as noted by the referee our results are significant and they may be even more so after following such filtration.

### Reviewer's Report 3

Reviewer 3: Emmanuel Levy, MRC Laboratory of Molecular Biology, Cambridge, UK (nominated by Sarah Teichmann, MRC Laboratory of Molecular Biology, Cambridge, UK)

#### Reviewer's comment

In this paper, Schweiger and Linial conduct an analysis of proximity, or clustering of phosphorylation sites within proteins. Using a large dataset of phosphosites, mostly characterized by large-scale phospho-proteomics methods, they show that phospho-serines, threonines, and to a lesser but significant extent tyrosines, appear closer to each other in proteins than would be expected by chance. Anecdotal and family specific descriptions of such a clustering have been described before, but this is to my knowledge the first general analysis, which makes the conclusions of this paper of general importance. The data on clustering of sites phosphorylated by the same kinase are especially exciting.

The authors find a very strong signal regarding the clustering of phosphorylation sites. Yet, the strength of the signal should be reassessed using a null model that takes into account disordered regions. The reason is the following: it is known that ~80% of phosphorylation sites are in disordered regions, although these correspond to only ~30% of the proteome. These proportions should thus be maintained during the randomization process. The following analogy will illustrate my point: if proteins were people and proteins were the planet, the conclusion would be that people are clustered on the planet - this is true, but it would be important to take into account the structure of cities (e.g., disorder) when making such a statement. Even when taking into account the structure of cities, some clustering patterns are likely to persist (e.g., think of Manhattan). Because the aim of this paper is to uncover an underlying organization of phosphorylation sites, it is critical to assess the extent to which the clustering observed simply results from phosphorylation sites being in disordered regions. Therefore, the null model should shuffle phosphorylation sites within proteins and maintain the number of them present in ordered and disordered regions.

#### Authors' Response

Thanks for the nice analogy on Manhattan and structures of cities. An even stronger example is the surprising observation that Tel-Aviv and Jerusalem are on the same planet. We performed another calculation of the background distribution, this time maintaining the number of residues in ordered and disordered regions, as suggested. While the new background distribution is indeed different than the previously calculated distribution, it is still significantly different than that of the real distribution. Therefore, all the relevant conclusions remain intact. The distribution for S/T and Y based on this new analysis is provided in Additional file 4).

#### Reviewer's comment

The same comment applies to the functional analysis; i.e., is the functional enrichment of proteins containing S/T clusters different from that corresponding to proteins enriched in disordered regions? To test this, a "universe" of proteins should be created that has the same distribution of disordered regions as that of phosphorylated proteins, and the GO analysis should be carried out on this "universe".

#### Authors Response

In the paper we do not conduct a general analysis of the GO annotation of phosphoproteins. Instead, we closely study a few selected proteins that are extreme to the phenomenon reported (i.e., enrichment in clusters of phosphosites). These proteins were investigated with the idea that the properties of this set (Additional file 1) may hint to some functional preferences. We actually avoided any statistical interpretation for such a protein set. We therefore feel that concerns on such a bias in protein functions are irrelevant to this case.

#### Reviewer's comment

The DisEMBL methodology was used to predict disordered regions. It could be good to use DISOPRED [65], as it would increase the fraction of sites that appear in disordered regions (DISOPRED yields ~80% of all phosphosites in disordered regions, while the numbers currently mentioned are "68% and 43% for phosphosites that are at a distance ≤ 4 and >4, respectively").

#### Authors' Response

The definition of 'disorder' is strongly dependent on the specific application at hand. A categorization of more residues to disordered regions might come at the expense of false identification. Moreover, despite numerous efforts, we encountered technical difficulties in activating DISOPRED for offline large-scale analysis. Therefore we chose to keep our current analysis.

#### Reviewer's comment

Interpretation of the clustering of phosphosites. I totally agree that clustering of phosphorylation sites is functionally relevant and important in many instances, as described in the paper, and as remarkably illustrated in [14]. Yet, (at least) another interpretation could explain this clustering and should be discussed. The recognition motif of particular kinases is often so degenerate that additional specificity mechanisms must be at play, such as binding of the substrate protein via another site, or a scaffold protein that itself binds the kinase and substrate. In both of these cases, the net result is a local increase of the kinase-substrate concentration, which could facilitate the phosphorylation of the biological site, but also the promiscuous phosphorylation of sites situated nearby. In such a scenario, the promiscuous phosphorylation would be expected to be less efficient, and thus the stoichiometry of phosphorylation would be expected to be lower. Such a scenario is supported by some of our results [28], where among pairs of phosphorylation sites close to each others, the one with lower stoichiometry is less conserved on average.

#### Authors' Response

The referee raised a valuable discussion and a present insight of a potential connection between stoichiometry and conservation. With the current limitations of quantitative measurements of phosphosite stochiometry, validation of the proposed scenario remains a technological challenge.

#### Reviewer's comment

Conservation of phosphorylation sites. I also wish to correct a mis-interpretation regarding the conservation of phosphorylated sites (interestingly this is not the first time that I notice this mis-interpretation, which is why I would like to put an emphasis on it). The authors cite our work [28] to support the notion that "the conservation rate of phosphosites [...] is a hotly debated topic", and the work of Soon Heng Tan et al [35] to support that "no specific conservation trend is assigned to pS/pT sites". However, there is no real contradiction between the results obtained by different research groups. We, like Soon Heng Tan *et al*. and others (e.g., [27,32] as cited in the paper) show that phosphorylated sites are significantly more conserved than equivalent but non-phosphorylated residues. However, "significantly" should not be mistaken for "a lot more". As a matter of fact, although the conservation is significant, it is not very different, which could be explained by (at least) two effects: (i) compensation mechanisms may be at play. In other words, if a function is linked to a cluster of sites rather than an individual site, then sites within the cluster may be relatively free to be lost and re-gained at nearby positions. This is actually very relevant to the idea of functional clusters put forward in this paper, and the authors could cite a recent paper by Holt *et al*. [37] to support it - it would also be more appropriate to cite the paper by Soon Heng Tan *et al*. [35] in that context, since their method allows one to study this mechanism. (ii) An additional effect, that could contribute to explain the not-so-strong conservation, is that a fraction of sites that are detected may result from promiscuous phosphorylation events [28].

#### Authors' Response

We have changed our statements that mention an apparent controversy for pS/pT/pY conservation. In the literature supportive evidence for 'lower than expected' conservation and for a fast evolutionary dynamics exists. We rephrase the discussion to account for the suggestions raised by the referee on the gain/lost dynamics of nearby sites. We included the relevant references and as proposed by the referee. We have not included the possible role of promiscuous phosphorylation events as we can not support this possibility with our present data.

#### Reviewer's comment

Dependence of the phosphorylation state of proximal sites. The idea that there is a dependency between the phosphorylation states of proximal sites is appealing and original. However I find it difficult to draw conclusions from the current analysis of the data, because no statistical test is performed to compare the frequency of occurrence of the R and L states against B states (I'm not sure if anything can be concluded regarding the None state since by definition, peptides without a phosphate group are generally not purified by current experimental setups). In other words, it would be helpful to guide the reader as to why the results presented in Fig. 5 allow one to conclude that B is indeed over-represented.

#### Authors' Response

Since the dataset detailing where phosphosites were found is more comprehensive than that dataset of actual peptides and their phosphorylation pattern, 'None' states are possible; a certain phosphosite can be reported in one report, while missing completely from all the peptides found from its protein in another report. On a more general note, while we indeed think that B is over-represented, the problem of assigning a correct P-value to an appropriate statistical model appears highly non-trivial. We agree that this is no replacement for a thorough, directed set of experiments that will enable a more rigorous analysis, as we detailed in the body of the paper itself. However we feel that this information is still worth presenting in spite of these drawbacks. We should also mention that phosphorylation peptide data are rapidly accumulating. We have been able to support the trends seen in Fig. 5 using several independent sets of large-scale phosphopeptide studies.

## Supplementary Material

Additional file 1 **Supplementary data S1**. List of exceptionally cluster-rich proteins and their functional assignments. Source data for Figure 4. Click here for file

Additional file 2 **Supplementary data S2**. Distribution of the number of possible protein kinases. Supportive information for Table 3. Click here for file

Additional file 3 **Supplementary data S3**. The distribution of the distance to the nearest phosphosite, for real phosphosites and random phosphosites; where the random distribution was calculated taking into consideration the actual number of sites on the protein (see Materials and Methods, and also Reviewers' Comments). Click here for file

Additional file 4 **Supplementary data S4**. The distribution of the distance to the nearest phosphosite, for real phosphosites and random phosphosites; where the random distribution was calculated taking into consideration the actual number of sites on the protein, and also the number of residues in 'ordered' and 'disordered' regions (see Materials and Methods, and also Reviewers' Comments). Click here for file

Additional file 5 **Supplementary data S5**. Extension of Figure 2A (see Reviewers' Comments). Click here for file
