# Supplementary material for: Cooperativity within proximal phosphorylation sites is revealed from large-scale proteomics data
Source: Biol Direct. 2010 Jan 26;5:6. doi: 10.1186/1745-6150-5-6 (PMC2828979; doi:10.1186/1745-6150-5-6)

**Supplementary data S2.**

Distribution of number of possible kinases (supporting data for Table 3).

| **No. of Possible Kinases** | **Site Count** |
| --- | --- |
| 1 | 18877 |
| 2 | 565 |
| 3 | 114 |
| 4 | 29 |
| 5 | 13 |
| 6 | 7 |
| 7 | 1 |
| 8 | 1 |
| 9 | 0 |
| 10 | 0 |
| 11 | 1 |


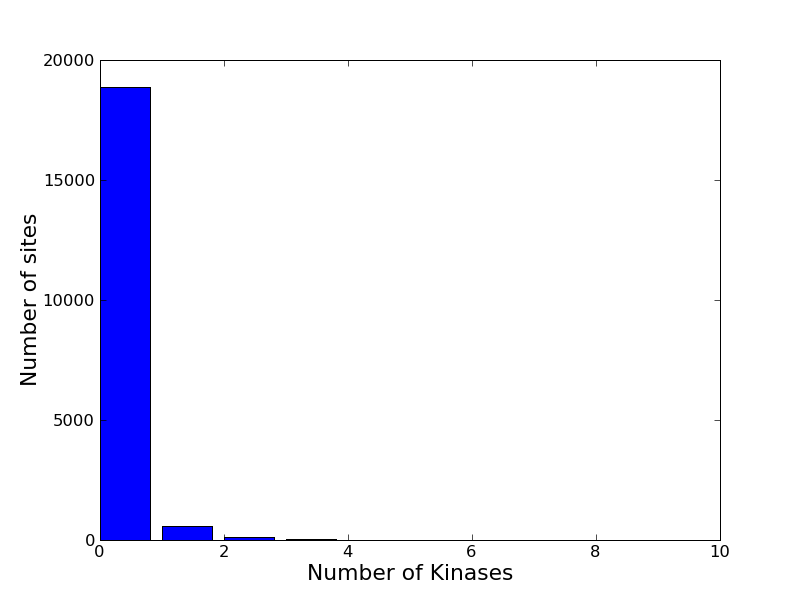

Supplement: Additional file 2 — Supplementary data S2. Distribution of the number of possible protein kinases. Supportive information for Table 3. [file 1745-6150-5-6-S2.DOC]
