# Supplementary material for: Cooperativity within proximal phosphorylation sites is revealed from large-scale proteomics data
Source: Biol Direct. 2010 Jan 26;5:6. doi: 10.1186/1745-6150-5-6 (PMC2828979; doi:10.1186/1745-6150-5-6)

**Supplementary data S3.**

The distribution of the distance to the nearest phosphosite, for real phosphosites and random phosphosites; where the random distribution was calculated taking into consideration the actual number of sites on the protein (see Materials and Methods). (A) for S/T sites; (B) for Y sites.

(A)


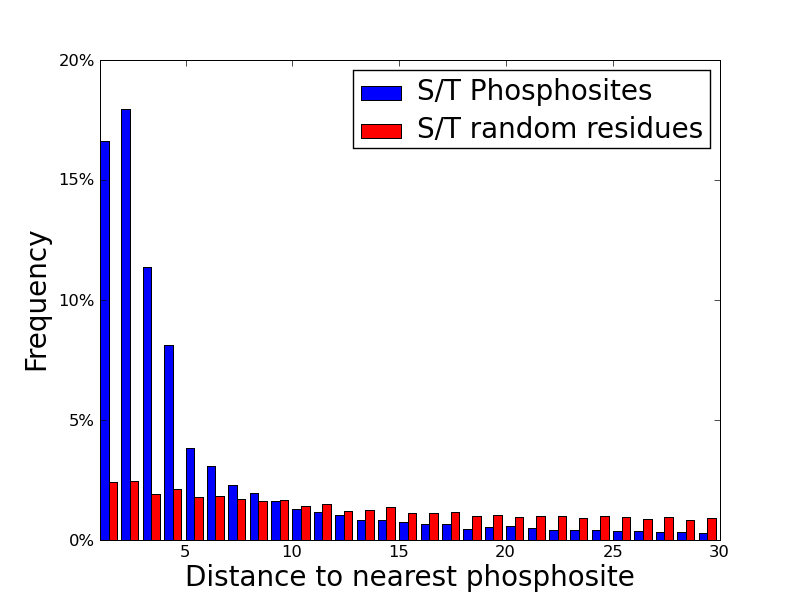


(B)


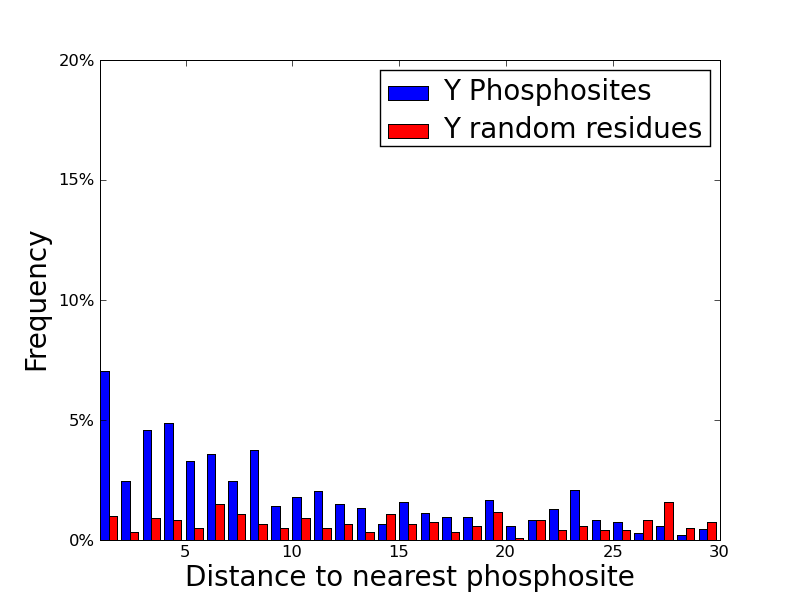

Supplement: Additional file 3 — Supplementary data S3. The distribution of the distance to the nearest phosphosite, for real phosphosites and random phosphosites; where the random distribution was calculated taking into consideration the actual number of sites on the protein (see Materials and Methods, and also Reviewers' Comments). [file 1745-6150-5-6-S3.DOC]
