# Supplementary material for: Cooperativity within proximal phosphorylation sites is revealed from large-scale proteomics data
Source: Biol Direct. 2010 Jan 26;5:6. doi: 10.1186/1745-6150-5-6 (PMC2828979; doi:10.1186/1745-6150-5-6)

**Supplementary data S4.**

The distribution of the distance to the nearest phosphosite, for real phosphosites and random phosphosites; where the random distribution was calculated taking into consideration the actual number of sites on the protein, and also the number of residues in ‘ordered’ and ‘disordered’ regions (see Materials and Methods). (A) for S/T sites; (B) for Y sites.

(A)


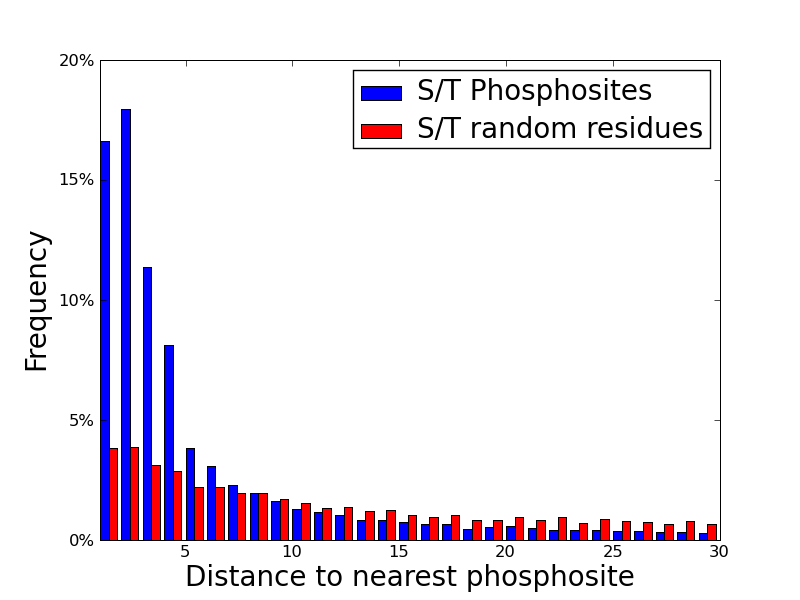


(B)


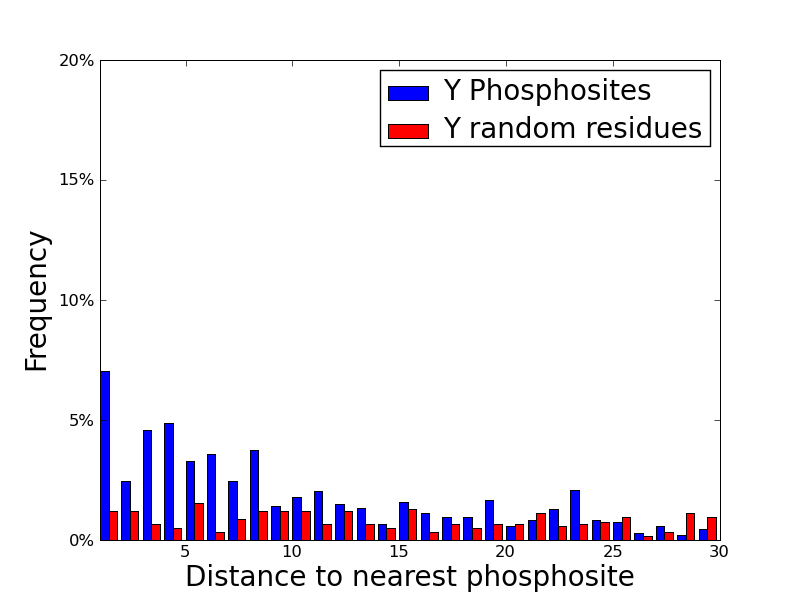

Supplement: Additional file 4 — Supplementary data S4. The distribution of the distance to the nearest phosphosite, for real phosphosites and random phosphosites; where the random distribution was calculated taking into consideration the actual number of sites on the protein, and also the number of residues in 'ordered' and 'disordered' regions (see Materials and Methods, and also Reviewers' Comments). [file 1745-6150-5-6-S4.DOC]
