# Supplementary material for: Cooperativity within proximal phosphorylation sites is revealed from large-scale proteomics data
Source: Biol Direct. 2010 Jan 26;5:6. doi: 10.1186/1745-6150-5-6 (PMC2828979; doi:10.1186/1745-6150-5-6)
Supplement: Additional file 5 — Supplementary data S5. Extension of Figure 2A (see Reviewers' Comments). [file 1745-6150-5-6-S5.DOC]

**Supplementary data S5.**

The distribution of the distance to the nearest phosphosite, for real phosphosites and random phosphosites – this data are partially shown in figure 2A, but the distance to nearest phosphosites was extended until the distance of 250 amino acids.
